# Impacts of chronic disease prevention programs implemented by private health insurers: a systematic review

**DOI:** 10.1186/s12913-021-07212-7

**Published:** 2021-11-11

**Authors:** Sithara Wanni Arachchige Dona, Mary Rose Angeles, Natasha Hall, Jennifer J. Watts, Anna Peeters, Martin Hensher

**Affiliations:** 1 Institute for Health Transformation, Faculty of Health, Deakin University, Geelong, Victoria 3220 Australia; 2 Deakin Health Economics, School of Health and Social Development, Faculty of Health, Deakin University, Geelong, Victoria 3220 Australia

**Keywords:** Chronic diseases, Private health insurers, Prevention

## Abstract

**Background:**

Chronic diseases contribute to a significant proportion (71%) of all deaths each year worldwide. Governments and other stakeholders worldwide have taken various actions to tackle the key risk factors contributing to the prevalence and impact of chronic diseases. Private health insurers (PHI) are one key stakeholders, particularly in Australian health system, and their engagement in chronic disease prevention is growing. Therefore, we investigated the impacts of chronic disease prevention interventions implemented by PHI both in Australia and internationally.

**Method:**

We searched multiple databases (Business Source Complete, CINAHL, Global Health, Health Business Elite, Medline, PsycINFO, and Scopus) and grey literature for studies/reports published in English until September 2020 using search terms on the impacts of chronic disease prevention interventions delivered by PHIs. Two reviewers assessed the risk of bias using a quality assessment tool developed by Effective Public Healthcare Panacea Project. After data extraction, the literature was synthesised thematically based on the types of the interventions reported across studies. The study protocol was registered in PROSPERO, CRD42020145644.

**Results:**

Of 7789 records, 29 studies were eligible for inclusion. There were predominantly four types of interventions implemented by PHIs: Financial incentives, health coaching, wellness programs, and group medical appointments. Outcome measures across studies were varied, making it challenging to compare the difference between the effectiveness of different intervention types. Most studies reported that the impacts of interventions, such as increase in healthy eating, physical activity, and lower hospital admissions, last for a shorter term if the length of the intervention is shorter.

**Interpretation:**

Although it is challenging to conclude which intervention type was the most effective, it appeared that, regardless of the intervention types, PHI interventions of longer duration (at least 2 years) were more beneficial and outcomes were more sustained than those PHI interventions that lasted for a shorter period.

**Funding:**

Primary source of funding was Geelong Medical and Hospital Benefits Association (GMHBA), an Australian private health insurer.

**Supplementary Information:**

The online version contains supplementary material available at 10.1186/s12913-021-07212-7.

## Introduction

Worldwide, 71 % (41 million) of all deaths each year are attributed to chronic diseases [1], with chronic diseases responsible for nine out of ten deaths in Australia [2]. Chronic or non-communicable diseases are illnesses that “tend to be of long duration and are the result of a combination of genetic, psychological, environmental and behavioural factors” [1]. Chronic diseases lead not only to deteriorating quality of life for individuals [3] but also cause an economic burden on individuals, health systems and wider society [4].

Governments and other stakeholders worldwide have taken a broad range of actions to tackle the key risk factors which contribute to the prevalence and impact of chronic diseases [5]. In Australia in 2011, it was estimated that 31% of the total burden of disease was potentially preventable through actions on 29 behavioural, metabolic, environmental and dietary risk factors [6]. Health insurers are one of the key stakeholders in health systems worldwide with the capacity to act on chronic disease prevention and management. While globally there are a number of different health insurance settings, such as private health insurance (PHI), employer-sponsored health plans, veteran’s affairs and public health insurance [7], our focus in this study is on the emerging role of private health insurers in chronic disease prevention [8].

Globally, prepaid private health spending, which included PHI, accounted for 17.4% of total health financing in 2014 [9]. This varied across countries. For example, PHI spending accounted for 6.6%, 7.1%, 38.3%, 44.2% of total health spending in New Zealand, UK, USA, South Africa respectively [9]. These differences reflect the fact that the scope, coverage and structure of PHI cover varies substantially between countries. Australia offers publicly funded universal health coverage for all citizens and permanent residents, through the national Medicare program and via the public health and hospital systems in each state and territory of the Federation. Yet PHIs play an important role in the Australian health system, accounting for some 9% of total health expenditure [10], especially in the funding of elective surgery and hospital care. However, in the Australian setting, PHI does not have full responsibility for the integrated care of insured members, most crucially because PHI is not permitted to fund primary health care and general practice [11]. Despite these constraints, interest in the role, incentives and ability of PHI to engage more closely in the prevention of chronic diseases is growing [12]. Action is growing not only in programs focused on primary and secondary prevention but also in self-management, which plays a crucial role in managing chronic diseases successfully to prevent further occurrence of symptoms or further complications [13]. Chronic disease management (CDM) activities focus on people with already-diagnosed chronic conditions. Despite legislation permitting PHI funds to pay benefits for CDM services having been in place for more than 10 years in Australia, insurers are still in an early stage of implementation and evaluation of CDM activities. During a qualitative study in 2019, PHI representatives reported identifying target groups; evaluating service outcomes; and collaborating with other healthcare providers as challenges to providing appropriate CDM programs [11]. Nonetheless, PHI’s role in chronic disease prevention and supporting people with chronic diseases to improve quality of life is emerging [11], through adopting chronic disease prevention interventions beyond the coverage of specific insurance products.

Previously there have been systematic reviews undertaken on financial incentives provided by both public and private health insurers for the prevention and management of overweight and obesity [7] and rapid or systematic reviews on chronic disease prevention interventions, but these were either focused on one kind of intervention or delivered by stakeholders other than PHI [14, 15]. Therefore, the aim of this paper is to systematically review to summarise the impacts of chronic disease prevention interventions implemented by PHI both in Australia and internationally, in order to provide lessons on how PHI might engage most effectively with primary, secondary and/or tertiary prevention of chronic diseases.

## Methods

### Search strategy

This systematic review is reported following Preferred Reporting Items for Systematic Review and Meta-Analysis (PRISMA guidelines) [16], 2020 checklist (Additional File 1). We conducted a systematic literature search across seven online databases: Business Source Complete, CINAHL, Global Health, Health Business Elite, Medline, PsycINFO (APS PsycINFO), and Scopus. Additional relevant literature and grey literature (i.e. reports) were searched using Google, Google Advanced, and reference lists of the included articles. Search terms were developed around PHI and chronic disease prevention or health promotion programs (see Additional File 2).

Studies were included if they evaluated the impacts of chronic disease prevention interventions implemented by PHIs. Primary, secondary and tertiary prevention interventions are defined respectively as reducing the incidence of disease before onset; preventing diseases at very early stage from progressing; and managing the manifest disease and its complication to improve quality of life [17]. Studies on the impacts of chronic disease management programs by PHIs were also included if prevention strategies were incorporated. Studies published in English until September 2020 were included. The search was not limited to specified study designs or a population. Studies were excluded if they were on impacts of programs primarily not implemented by PHI; or programs implemented by government funded public health insurers; or programs implemented by workplaces/employers with no sponsorship from PHI; or chronic disease care/treatment programs with no prevention strategies involved; or studies about expanded private health insurance coverage for various health conditions; or value based insurance designs (i.e. programs that provide reduced prescription drug co-payment costs for targeted chronic diseases). Studies published in languages other than English, abstracts, conference papers, newspaper articles, and study protocols were excluded.

### Study selection and data extraction

The selection of studies during the screening process was based on the above eligibility criteria. Two reviewers (SWAD and NH) first screened all the titles and abstracts independently using EndNote software. Selected papers were then divided equally between the two reviewers (SWAD & NH) for full-text screening and cross-checked. Any disagreement in either of these steps was discussed with one other author of this paper (MH) to reach a consensus. If full text articles were not found, they were retrieved by contacting Deakin University librarians via inter-library loan requests. Two reviewers (SWAD and NH) independently extracted the following data on an Excel table: country, program name, description of the program, health insurer, target population (community/members), study design, time period of the study, sample, any outcome measure and results. The literature was synthesized thematically; no meta-analysis or statistical analysis was conducted due to wide heterogeneity of the intervention types and outcome measures reported in this literature. As this review is exploratory in nature, we summarised any outcomes that have been used in this area and effect measures as reported in studies. Summary results of the studies are provided in tabular forms in the text and as additional files. Given the heterogeneity and observational nature of studies, we used the available evidence as possible and did not attempt to assess the reporting bias or certainty of outcomes.

### Risk of bias and quality assessment

For assessing risk of bias of included studies, two reviewers conducted the quality assessment independently (SWAD and MR). The review team discussed and resolved any discrepancies in the overall quality rating. The quality of selected studies was checked using the quality assessment tool for quantitative studies developed by Effective Public Healthcare Panacea Project (EPHPP), Canada [18]. We considered a range of tools for the assessment of risk of bias, including CASP, NIH, and STROBE; EPHPP was chosen due to the variety of methods and the quantitative nature of the included studies. The tool includes six components to rate study quality as Strong, Moderate or Weak. Studies that were rated strong were considered to have a low risk of bias, while weak studies were considered as having a high risk of bias.

## Results

### Search results

**Fig. 1:**
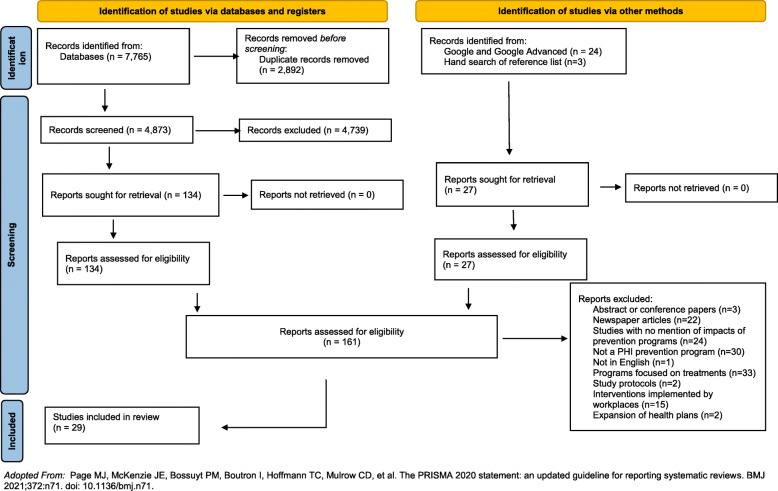
Consort chart

A total of 29 studies were included in the final thematic synthesis (Fig. 1). Table 1 provides the characteristics of the included studies. The majority of the studies were from the USA (*n* = 16, 55%) followed by South Africa (*n* = 6, 21%). The other countries were Australia (*n* = 5) and Germany (*n* = 2). Four studies were randomised controlled trials (RCT), but the majority of the studies were cohort studies (*n* = 14, 48%). Other study designs were cross sectional (n = 6), case control (n = 2), mixed method (n = 1), a pre and post evaluation study (n = 1), and a systematic review (n = 1).

**Table 1:** General Characteristics of the included studies

Author, year	Country	Private Health Insurance company	Target population	Program	Duration of the intervention considered for the study	Study Sample	Study type	Outcome measure
***Studies on financial incentives***								
An et al., 2013	South Africa	Discovery	Members	Cash-back for healthy food program	Feb 2009 to Nov 2011	350,000 participants and non-participants	Retrospective cohort	Self-reported measures of food consumption and weight status (BMI)
Patel et al., 2011	South Africa	Discovery	Members	Vitality health promotion program	2001–2006	*N* = 192,467 participants Mean age = 44 years % female = 35%	Retrospective cohort	Changes in the participation in activities on fitness, and hospital admissions
Patel et al., 2010	South Africa	Discovery	Members	Vitality physical activity	1 year (2006)	948,974 members	cross-sectional	Hospital admissions
Sturm et al., 2013	South Africa	Discovery	members	Cash-back for healthy food	Nov 2009 – March 2012	100,344 (intervention) 69,141 (control)	Case control	Changes in healthy food purchases
Hubbert et al., 2003	USA	University of Alabama at Birmingham’s (UAB)-owned HMO	Members	Financial incentives on a weight loss program	January 1998–February 2001	*N* = 125 participants Mean age = 49 years % female = 67%	Cohort with matched controls	Participation rate and weight loss
Schwartz et al., 2014	South Africa	Discovery	Members	Financial incentives	January 1, 2012 to June 30, 2012	6570 households Age and gender profile not provided	RCT	Healthy food purchases
Ball et al., 2017	Australia	GMHBA	Members	the ACHIEVE (Active Choices IncEntiVE)	June-Nov 2015	80 participants %female = 56% Men age no provided	Cohort- Pre-post design	Changes in physical activity and sedentary behaviour
Lambert et al., 2009	South Africa	Discovery	Members	Vitality – incentive based physical activity	1 year (2006)	948,976 members	Cross sectional	Hospital admissions and costs
McGill et al., 2018	Australia	HCF and other two unknown insurers	Members	Australian weight loss and lifestyle modification program (Financial incentives)	18 weeks, 2015	130 and 28 participants for 2 samples from intervention %female = 67.7 and 46.6%	Mixed method	Attitudes and views of participants
***Studies on health coaching***								
Adams et al., 2013	USA	Kaiser Permanente	Members	Health coaching	6 month period in 2011	*N* = 1410 members who had initial session between Jan 1, 2011, and Aug 23, 2011	Cross- sectional survey	patient satisfaction and perceived success
Koocher et al., 2001	USA	Fallon community health care	Community	Medical crisis counselling	Not provided	20 participants (intervention) 18 (control) Mean age = 57.5 Gender profile = not provided	RCT	Reductions in distress, hospital utilisation and costs
Härter et al., 2013	Germany	KKH-Allianz	Members	Health coaching	3 months (2007)	834 participants Mean age- = 66.2% female = 58.3%	Cross-sectional study	Evaluation of the coaching and process, effects on patient and physician communication
Schwartz et al., 2010a	USA	Highmark Inc., Blue Cross Shield	Members	Online disease management program with coaching	2004–2006	413 participants (intervention) 360 (control)	Retrospective quasi experimental cohort	Medical costs and claims
Scuffham et al., 2019	Australia	Bupa	Members	Health coaching for disease management (CAPICHe)	1 year (2012)	35,535 participants (intervention) 8883 (control) Mean age = 72.7 (intervention & control) %female = 46% (intervention) 46.2% (control)	RCT	Healthcare utilisation and costs
Lawson et al., 2013	USA	Not provided	Members	Health coaching-telephonic	January 1, 2009 to December 31, 2010	570 participants	Cohort study	Health and behavioural outcomes
Schmittdiel et al., 2017	USA	Kaiser Permanente	Members	Wellness coaching on weight loss	12 months (January 1 to August 232,011)	954 participants (intervention) 19,080 (control) Mean age = 52%female = 83%	Cross sectional	Changes in weight loss
Harmar et al., 2010	Germany	A German private insurance company	Members	Telephonic coaching in Chronic care management program	January 2, 2008 to January 1, 2009	17,319 participants (intervention) 5668 (control) Mean age 71.2 (intervention) Mean age = 72.5 (control) % Female = 50.8 (intervention) % female = 45.6	Retrospective quasi experimental cohort	Hospital admission rate
Morello et al., 2016	Australia	An Australian Private Health Insurer	Members	Telephonic Complex Care Program	6 months (2012)	273 participants (intervention) 232 (control) Mean age = 79 for both groups % female = 57.5% (Intervention) % female = 59.1% (control)	Case control	Hospital use and benefits paid
***Studies on wellness programs***								
Harris, 2011	USA	Blue shield	Members	Wellness program (Healthy Lifestyle rewards program)	2 years (2006 and 2007)	8003 (intervention) 187,631 (control)	observational cohort study	Medical utilisation, health claims, health risk behaviours
King et al., 2012	USA	Not provided a particular name	Members	Health-plan sponsored fitness centers	2012	Ranged from 618 to 4766 participants 1413 to 9035 for control	Systematic review	Changes in physical activity and positive health outcomes, health utilisation
Maeng et al., 2013	USA	Geisinger Health Plan	Members	MyHealth Rewards (health plan-driven employee health and wellness program)	2007–2011	Geisinger health plan employees as intervention and members as control	Cohort	Impact on cardiovascular event rate and health costs
Henry et al., 2016	USA	Kaiser Permanente	Members	Internet based health promotion	from December 2014 to March 2015	838,638 participants Mean age = 49.5% female = 48.9%	Retrospective cohort	Reducing care gaps on haemoglobin A1c testing, pneumonia vaccination, and cancer screenings
Frost et al., 2018	USA	Kaiser Permanente	Community	Physical activity	2014–2015	148 participants No gender profile at individual level % female at school level = 50%	Pre and post evaluation study	Participation in physical activity and changes in physical activity
Cheadle et al., 2018	USA	Kaiser Permanente	Community	Healthy eating, Active living	2011–2015	1300 residents from HEAL Zones (intervention) 1300 residents from outside of HEAL Zone (Control)	Cohort -pre and post design	Changes in food and physical activity behaviours
McGill et al., 2020	Australia	Not specified (In PHI setting)	Members	The Healthy Weight for Life (HWFL)	August 2019–August 2020	490 participants	Cohort- Pre and Post design	Changes in anthropometric and lifestyle risk behaviours
Coombes, 1998	USA	Kaiser Permanente and Oxford health Plan	Members	Malnutrition screening	Not provided	Not provided	cross-sectional survey	Identification of risk patients
Schwartz et al., 2010b	USA	An independent licensee of the Blue Cross and Blue Shield Association (Hawaii Medical Service Association)	Member	wellness and disease prevention program (HealthPass)	2002–2005	11,883 participants (intervention) 154,327 (control) Mean age = 51.7 50.7 (control) %female = 58.3% (intervention) 52.3% (control)	Retrospective cohort	Healthcare costs
***Studies on group medical appointments***								
Beck et al., 1997	USA	Kaiser Permanente	Members	Group outpatient medical appointment	1 year	321 participants with chronic diseases (intervention groups = 160 and usual care = 161) Mean age = 72 (intervention) Mean age = 75 (control)	RCT	Health service utilisation and cost, self-reported health status, and satisfaction
Hinchman et al., 2006	USA	Kaiser Permanente	Community	Childhood obesity	8 weeks to 6 months (2001–2003)	135 participants % Female = 55% Mean age not provided Aged from 11 to 17 years	Retrospective cohort	Body fat reduction, waist size, BMI

Twelve studies (41%) looked at the impacts of primary prevention measures delivered by PHI to reduce chronic diseases. Two studies evaluated the impacts of secondary prevention measures, and eleven studies were based on tertiary prevention measures. Three studies evaluated programs that incorporated mixed strategies of prevention levels such as both secondary and tertiary prevention (Table 2). The majority of the programs were for PHI members (*n* = 28, 93%) while the others were at community level. Table 2 provides the description of interventions evaluated in the studies.

**Table 2:** Description of chronic disease prevention programs

Author, year	Health Insurance company	Program	Program Description	Prevention Level
***Studies on financial incentives***				
An et al., 2013	Discovery	Financial incentives-Cash-back for healthy food	The HealthyFood program offers members up to 25% cash back on healthy food purchases on an ongoing basis which has spread nationwide across South Africa with about 260,000 enrolled households and 800 supermarkets.	Primary prevention
Patel et al., 2011	Discovery	Vitality health promotion program	From 2001 to 2003, with a $15 monthly family fee, participants are offered with a subsidised gym membership and access to other fitness centre chains, and receive points as discounts on good and services at 20 and 40%.	Primary prevention
Patel et al., 2010	Discovery	Vitality physical activity	In 2006, the Vitality program was based on activities on fitness, screening and health assessment, education and healthy choices. Cost for a family was about $10. Participation allowed to gain points which could be used as discounts from 15 to 45% for a range of purchases and services.	Primary prevention
Sturm et al., 2013	Discovery	Cash-back for healthy food (positive cash reimbursement)	Ongoing incentive program. Study was conducted over 31 months. Participants had an average of 10.9 months on 10% rebate and 14.3 months with the 25% rebate.	Primary prevention
Hubbert et al., 2003	University of Alabama at Birmingham’s (UAB)-owned HMO	Financial incentives on a weight loss program	Incentives were given for a group lifestyle based weight management program for 12 weeks. If one lost 6% or more of their initial weight and participated in more than 10 sessions, $150 was received as reimbursement at the end of the program which was a half of the program fee paid by the member. EatRight weight management program which included mean plans with controlled calorie	Primary prevention
Schwartz et al., 2014	Discovery	Financial incentives (negative cash reimbursement)	Monthly HealthyFood cash-back bonus from Vitality program for a duration of 6 months was implemented and the incentive was $113. The cost of groceries was $56 per month per family to join the program. Participants also risk losing the previously received reimbursement. Monthly email feedback about % of healthy items bought compared to baseline	Primary prevention
Ball et al., 2017	GMHBA	the ACHIEVE (Active Choices IncEntiVE)	Financial and non-financial incentives were given ranging from AUD$7.50 to $50 each (total value $193.50 for women and $196.50 for men); and a chance to win one of four Apple iPad Mini devices (lottery-based incentive), worth $454. The intervention rewarded positive behaviors, motivating to increase physical activity and reduce sedentary behaviour, with the ultimate aim of achieving 150 min of physical activity per week, and a reduction of 150 min per week of sedentary time.	Primary prevention
Lambert et al., 2009	Discovery	Vitality – incentive based physical activity	Vitality program was offered to members at about $12 per family. The sample was members whose incentives had been effective for 12 months in 2006.	Primary prevention
McGill et al., 2018	HCF and other two unknown	Australian weight loss and lifestyle modification program (Financial incentives)	The Healthy Weight for Life program delivered three six-week phases over 18 weeks for those with weight related chronic diseases. The phases included eating plans with controlled portion, recommendations for physical activities and tracking progress, motivation, support and advice through SMS, phone, mail and emails.	Tertiary prevention
***Studies on health coaching***				
Adams et al., 2013	Kaiser Permanente	Health coaching	A health coaching program through centralised telephonic Wellness Coaching Center across Kaiser Permanente Northern California which includes 48 medical facilities since January 2010. This was basically focused around motivational interviewing, which was a patient centred counselling style. This included one initial session for 20–20 min and a 10–20 min follow-up session. Coaching provided support with five lifestyle changes: healthy eating, physical activity, weight management, tobacco use cessation, and stress. Coaches record the session in participants’ electronic medical record.	Tertiary prevention
Koocher et al., 2001	Fallon community health care	Medical crisis counselling	Medical crisis counselling service was offered for patients diagnosed with cancer to prevent related psychological issues. Patients were offered up to 10 sessions. The program focused on supporting to cope with the disease and distress. Patients charged $2 per visit co-payment fee for each session.	Tertiary prevention
Härter et al., 2013	KKH-Allianz	Health coaching	Health coaching was offered for members with one or several chronic diseases. The coaching included goal attainment, information in medical conditions. The program is customised based on individual member such as monitoring weight, coaching on therapy adherence, and influenza vaccination.	Tertiary prevention
Schwartz et al., 2010a	Highmark Inc., Blue Cross Shield	Wellness program through an online disease management program	A Health Promotion and Disease Prevention Program was implemented for employees and members that included a set of online options, with health risk assessment, and online programs focused on managing healthy lifestyle, fitness, nutrition and stress, and tobacco cessation. This was part of the Blues on Call program, which is available for people with one or more chronic conditions. Telephone counselling delivered by registered nurses and dietitians was included, with interactive voice response telephonic outreach to members, letter and phone call reminders for clinical preventive exams, educational resources. Program began in September of 2004, along with an online chronic condition self-management intervention (HealthMedia® Care for Your Health) that works in conjunction with Blues on Call health coaches to help members manage chronic conditions.	Tertiary prevention
Scuffham et al., 2019	BUPA	Coaching Health (CAPICHe) trial	Health coaching trial included programme awareness notifications, health Coaching session, and follow-up calls (from one call in the first 2 months with no maximum number of calls being set), accessing to health coaches via telephone as required and tailored outreach and educational materials.	Tertiary prevention
Lawson et al., 2013	–	Health coaching-telephonic	Health coaching program was focused om health behaviour change, motivating and educating about self-management of the disease. The program did not focus on diagnosis, treatment or complications or symptom management, but on the physical, mental and spiritual aspects of wellbeing.	Tertiary prevention
Schmittdiel et al., 2017	Kaiser Permanente	Health coaching	The health coaching program was for weight management, physical activities and healthy eating behaviours. The first session was for 20–30 min for speaking to coach on the telephone, deciding on a health topic to focus, assessing readiness to change, and choosing an action step to begin. Follow-up sessions are about 10–15 min based on request and should complete within a year or less.	Tertiary prevention
Harmar et al., 2010	German private insurance company	Chronic care management	Chronic disease management program was initiated in 2008 which is patient-centred services that focused on the full scope of chronic conditions, risk factors, and behaviours to support patients to treat the condition and manage their health. The program focused on educating and empowering members such as health-related behaviours, measures on self-care, and adherence to care. This was mainly facilitated by telephonic call support by nurses)	Tertiary prevention
Morello et al., 2016	An Australian PHI	Telephonic Complex Care Program	The program was implemented on members with more hospital admissions. Program cost were included in the insurance membership. After a health assessment of risk, members were then offered with telephone support over six months, personalised care plan and referral to community based services. After the phone calls, a letter was sent which included information discussed in the call.	Tertiary prevention
***Studies on wellness programs***				
Harris, 2011	Blue shield	Wellness program (Healthy Lifestyle Rewards program-HLR))	An online wellness program on modifiable risk factors such as physical activity, healthy food, smoking prevention and cessation, and stress. The program includes registration the HLR website, and completion of a health risk assessment (HRA) or Wellness Assessment questionnaire. Supplemental components of include logging into the HLR website at least once weekly and completing a wellness activity, and an on-site biometric screening of height, weight, among other markers. Participants can earn up to $200 per year for participating. An individual earns $50 for completion of the Wellness Assessment (step 2), and an additional $50 for every 12 weeks logging into the HLR website and completes a wellness-related activity.	Primary prevention
King et al., 2012	–	Coverage of fitness centre membership (Fitness Sponsorship)	This systematic review was on health-plan sponsored fitness centre membership benefit (Known as the Silver Sneakers program)	Primary prevention
Maeng et al., 2013	Geisinger Health Plan	MyHealth Rewards (health plan-driven employee health and wellness program)	Wellness program stated in July 2006. It included health risk assessment, Employees with the health plan membership were eligible for medications for hypertension, high cholesterol, and diabetes with $0 co-pay to the employee, structures disease management programs along with financial incentives to participate. Participants received $200 incentive for enrolment and addition $200 at 6 months, and another $200 after one year completion. The program was strategies for self-management, nutrition and physical activity, medication management in collaboration with the employee’s primary care providers, and acute exacerbation management.	Tertiary prevention
Henry et al., 2016	Kaiser Permanente	Internet based health promotion	Kaiser Permanente Southern California offered members an online Personal Action Plan with a web portal to access to information about prevention, health promotion, and care gaps.	Secondary prevention
Frost et al., 2018	Kaiser Permanente	Physical activity	Kaiser Permanente Colorado’s community health initiated increased access to healthy eating and active living in LiveWell Colorado. Under this, a playground in intermediate schools were redesigned and additional equipment were added to encourage physical activities. The study focused on monitoring students at school before redesign in 2014, at 6 months and 12 months after the redesign.	Primary prevention
Cheadle et al., 2018	Kaiser Permanente	Healthy eating, Active living	The healthy Eating Active Living (HEAL) Zones obesity prevention initiative was delivered in 2011–2015 in 12 low-income communities in Kaiser Permanente’s Northern and Southern California regions. HEAL Zones included policies, environmental and programmatic strategies. For example, Providing a new physical education curricula in kindergarten to 12th-grade schools, installing a lighted walking trail to provide access to safe physical activity, healthy menus in restaurants, or media campaigns on health education. The main difference between these two regions is the duration where Northern area had two large scale interventions with longer duration (one from 20,006 to 2010 and the other is from 2011 to 2014). The Southern region had the first phase of the program from 2012 to 2016. Second phase started in 2016.	Primary prevention
McGill et al., 2020	Not specified (In PHI setting)	The Healthy Weight for Life (HWFL)	The program is an 18 week intensive weight loss and lifestyle modification program for those members who have BMI > 28 kg/m2 and those with a chronic disease. The program is offered remotely via phone, SMS, email, mail and online portal. The focus of the program is to support loss weight with controlled food portions, healthy eating for the first 12 weeks and recommendations for physical activities.	Primary and Tertiary prevention
Coombes, 1998	Kaiser Permanente	Malnutrition screening	Oxford initiated the Nutrition screening program to identify at-risk members and managed them via both clinical and non-clinical interventions such as access to a nutrition visit from a contracted dietitian and follow-up visits.	Secondary prevention
Schwartz et al., 2010b	An independent licensee of the Blue Cross and Blue Shield Association	wellness and disease prevention program (HealthPass)	HealthPass is a disease prevention and health promotion program implemented from 2002 to 2005 and started with a health risk assessment which assessed the member for lifestyle, habits, and health risks. Members also screened for biometric measures such as BMI, blood pressure, cholesterol, glucose. Other screenings such as pap smear were offered based on individual needs. There was a counselling session for each participant to discuss risks, wellness goals and changes in lifestyle. They were conducted individually or as groups or over the phone. Online wellness intervention program was implemented in the final 2 years of the program on managing weight, nutrition, stress and smoking cessation.	Primary and secondary prevention
***Studies on group medical appointments***				
Beck et al., 1997	Kaiser Permanente	Group medical appointment	Monthly group visits for one year. The groups visit included a 15 min warm up and socialisation, 30 min health related information presentation, 15 min of reviewing individual patient’s medical records, blood pressure readings, determine any care needs by nurses and attend patients when necessary by a physician. Then, another 15 min for questions and answers session. No changes made for the usual care patients.	Tertiary prevention
Hinchman et al., 2006	Kaiser Permanente	Childhood obesity	Operation Zero is a referral program for at risk of overweight or overweight preadolescents and adolescent patients. This involved group medical appointments with weekly one-hour appointments for two months and another four appointments at three months intervals. The program was family based and included health education, activities and healthy recipes.	Secondary prevention

A summarised quality assessment of studies is presented in Additional File 3. The majority of studies (*n* = 24, 83%) were scored as having an overall weak quality in methodology according to the quality assessment tool for quantitative studies developed by EPHPP. Four studies were of moderate quality and only one study was rated as strong quality.

### Impacts of PHI chronic disease prevention programs

This review identified four main categories of chronic disease prevention interventions being delivered by PHI. The selected studies were summarised based on these types: Financial incentives, health coaching, wellness programs, and group medical appointments. The outcomes and effect measures are varied across studies as summarised in this section (see Additional File 4).

### Financial incentives

Nine studies were based on the impacts of financial incentives and focused on primary prevention [19–27]. Six studies evaluated Vitality program (Healthy Food program) by Discovery Health, which is one of the largest PHI (or “medical aid”) funds in South Africa. Table 2 provides a description of the program. Eight of the studies were high risk of bias [19–24, 26, 27], and one was moderate risk of bias [25]. According to Sturm et al., 10% and 25% discounts on healthy food choices led to an increase in healthy/total food ratio of 6% and 9.3% as well as in fruit and vegetables/total food ratio by 5.7% and 8.5% respectively. A decrease in the ratio of less desirable/total food expenditure by 5.6% and 7.2% respectively [23]. A repeated survey of 350,000 participants and nonparticipants of HealthyFood program, which was part of Vitality, found out that subsidies on healthier foods significantly increased the purchase and consumption of promoted products [20]. Their study showed that discounts were associated with statistically significant positive changes in self-reported dietary behaviours compared to no discounts (*p* < 0.0001). Larger discounts were always associated with a larger effect compared to smaller discounts. Daily fruits and vegetable consumption increased by 40% and 60% for 10% and 25% discounts respectively. People (who receive 10% or 25% rebate) were twice and three times more likely to have three or more servings of wholegrain foods on a daily basis than no rebate. Similarly, people were 25% (10% discount) and 65% (25% discount) less likely to have foods high in sugar, as well as 40% (10% discount) and 75% (25% discount) less likely to have foods high in salt [20].

A longitudinal study on the incentivised Vitality program found a significant reduction in the prevalence of physical inactivity from 76 to 68% over a 5-year period, which is a risk factor for chronic diseases. Increased physical activity was associated with reduced probability of hospital admissions due to chronic diseases [21]. Members who were highly active within the first 3 years had less hospital admissions in year 4 to 5 (20.7%) compared to inactive members (22.2%). Gym use increased from 27 to 33.1% over the 5-year period and for each two extra gym visits per week the likelihood of a hospital admission was reduced by 13% (OR = .87; 95% CI 0.801, 0.949) [21]. Participants who were in the high active Vitality group (greater than 48 gym visits per year) had 35% less cancer and mental illness admissions than members in other groups. Admissions associated with endocrine, nutritional, metabolic disorders, kidney and urinary tract disorders were 20% lower compared with members in other activity groups. Admission rates were also 7.4% lower for cardiovascular disease, 13.2% lower for cancers [22].Another incentive based 4-month physical activity program, the ACHIEVE study, recruited inactive individuals aged 40–65 years via GMHBA in Australia and found that there were large increases in leisure time physical activity by 212.1 mins/week in men from 106.7 ± 135.1 to 318.8 ± 263.6 (*p*-value < 0.001) and 281.6 mins/week in women from 81.4 ± 105.3 to 363.0 ± 486.7 (*p*-value < 0.001); transport related physical activity by 139.6 mins/week in men from 73.4 ± 85.8 to 213.0 ± 223.3 (p-value < 0.001) and 207.1 mins/week in women from 81.2 ± 94.9 to 288.3 ± 371.4 (p-value < 0.001). Sitting time reduced by 3.1 h/day for both genders from 8.6 ± 2.6 to 5.5 ± 1.9 for men (p-value < 0.001) and from 8.4 ± 2.4 to 5.3 ± 2.1 for women (p-value < 0.001). There were also reductions in BMI by 1.3 kg/m2 (p-value < 0.001) and systolic blood pressure by 5.1 mmHg [25].

Regarding the long term effects of incentives, after a weight loss and lifestyle modification program within three Australian insurance companies, participants of the program reported non-cash (i.e. incentive with a monetary value but not cash) (85.2%) and cash (77%) incentives would be a motivation, but 40.5% reported deposit contracts (i.e. making a monetary deposit which is refunded if weight loss is maintained) would motivate maintaining weight loss [27]. 93.9% of respondents supported the idea of a maintenance program following the weight loss program. More than half of respondents were positive about providing financial incentives 3 to 6 months after the program. The majority (85.2%) thought non-financial incentives would motivate maintaining weight loss [27].

### Health coaching

Nine studies looked at the impacts of health coaching delivered by PHI [13, 28–35]. Eight studies were high risk of bias [13, 28–32, 34, 35], and one was moderate risk of bias [33]. In a health coaching program delivered by Kaiser Permanente, members who attended two or more sessions reported improvements compared to prior session they attended, such as having more healthy eating (68% vs. 54%, *p* = 0.04), increased physical activity (71% vs. 45%, p = < 0.001), improved health (79% vs. 61%, *p* = 0.005), improved quality of life (83% vs. 61%, *p* < 0.001), and reduced risk of diseases (73% vs. 51%, *P* = 0.10) [28]. Similarly, Lawson et al. found that telephonic health coaching significantly improved stress levels, healthy eating, exercise levels, physical and emotional health by 11.9%, 12.8%, 20.9%, 11.6% and 15.4% respectively [30]. General health status and quality of life was improved in all participants.

A randomised control study in a US Health Management Organisation (HMO) found that well-integrated mental health clinicians offering Medical Crisis Counselling (MCC) straight after a distressing diagnosis, (e.g., cancer or heart attack) found significant reductions in stress, anxiety and depression, increased patient satisfaction and increased perceived social support. Over the 6 months of the study, no increases in overall medical costs were found but some decreased mental health utilization and costs were noted with MCC use. Favourable patient outcomes were seen regardless of the number of MCC sessions and therefore the cost-efficiency of the MCC approach was described by the authors as “impressive” [29].

Schwartz et al. also looked at cost impact of Blues on Call program with telephone outreach to members with chronic disease started by Blue Cross Blue Shield in Pennsylvania. An economic evaluation found the health care costs per person per year were $757 less than predicted for participants undertaking ‘Blues on Call’ compared with matched non-participants. This equated to a return on investment of $9.89 for every dollar spent on the program [35]. The intervention group had significantly lower same-day admission costs and fewer same-day admissions per 1000 person-years. The health coached group also had significantly fewer hospital admissions for patients with COPD and fewer same-day admissions for patients with diabetes. The Blues on Call program also had significant differences in service utilisation for total number of services, professional services, and pharmacy services (*p* < .05). Patients had fewer total inpatient days and shorter length of stay per person per year and showed a declining trend from 2005 to 2007 [35]. However, Bupa Australia reviewed their health coaching program and found the total cost 1 year post-randomisation of health coaching compared to standard care was not significantly different [34]. Harmar et al. reported on a chronic disease program which incorporated telephonic outreach by nurses to members regarding self-care measures and educating on health-related behaviours and adherence to care by a German PHI [32]. After one-year of the intervention, the hospital admission rate decreased by 6.2% for the intervention group compared to the control group with a 14.9% increase (*p* < 0.001). The admission rate was recorded to be lower with but the number of telephone calls higher (*p* = 0.004) [32]. Nonetheless, another evaluation of the telephonic complex care program over 6 months in the Australian context (no name given for the PHI) found there was no significant difference in the number of hospital admissions or total hospital days between both participants and non-participants in the 12 months after the intervention [33].

Moreover, a study on health coaching by German PHI, KKH-Allianz, found that 78% of members were satisfied with coaching and 82% would recommend it to others [13]. More than half the participants discovered new options, via health coaching, to influence their health condition [13].

### Wellness programs

There were nine studies on wellness programs delivered by PHIs [36–44].Eight studies are high risk of bias [36, 37, 39–44], and one study is moderate risk of bias [38]. One US study examined the impacts of the Blue Shield California’s Internet based Wellness program (Healthy Lifestyle Rewards) and participants in wellness programs exercised more frequently, consistently ate more fruits and vegetables, and reported lower stress levels. They revealed that it was associated with statistically significant declines in all measures of outpatient utilization (including non-hospital physician and non-physician visits, and physician visits) in the following year. There were no significant effects on claims spending for cholesterol and depression medications, or in utilization in the categories of emergency department visits, and inpatient admissions [37].

Schwartz and colleagues evaluated a 4 year wellness and disease prevention program called ‘HealthPass’ and estimated a net savings of $374, $34, $132, and $124 per year per participant yielding an estimated return on investment of $2.83, $1.16, $1.56, and $1.58 for every dollar invested for the years of 2002–2005, respectively [44]. Similar to the health care expenditures, non-participants made more claims for inpatient and pharmacy services (*p* < 0.0001 for all years) compared to participants, who made more claims for medical services (p < 0.0001 for all years). The outpatient claims were not significantly different between the two groups (*p* > 0.10 for all years). HealthPass participants consistently had fewer inpatient days per year and shorter average length of stay per admission compared to non-participants. Another study on ‘MyHealth Rewards’, an employee health-plan driven health and wellness program in the USA, showed that on average program participants experienced heart problems later than non-participants did (*p* < 0.01). This corresponded with a 10 to 13% cost reduction during the 2nd and 3rd year of the program [40]. Similarly, a systematic review on health-plan sponsored fitness centres showed a significant healthcare cost saving after a 2 years through reduced hospital admissions ($500; 95% CI $892 to 106, P=0.01) compared to a 1 year which had no difference between participants and non-participants of the program [39].

Another wellness intervention by PHIs was screening programs. Oxford Health Plan initiated a nutrition screening program to screen at-risk members and offer interventions related to nutrition such as visits from a dietitian and follow-ups. This program was able to reduce the nutrition risk and led to reduction in the number of insurance claims and emergency department visits, resulting in a 538% return on the investment [41].

Coombs et al. indicated that at a cost of only 21 cents per member per month for malnutrition screening, they found reduced insurance claims, reduced claim dollars, and reduced ED visits after implementation of nutrition screening [41]. Another approach to wellness was the Kaiser Permanente Online Personal Action Plan (oPAP), using the member web portal to better enable members to access information about prevention, health promotion, and care gaps. Users of oPAP were more likely than non-registered members to close healthcare gaps, especially cancer screening tests [38].

As a community initiative, Kaiser Permanente updated community playgrounds and found that children engaging in moderate to vigorous physical activity during recess increased by 23.3% (*p* < 0.01), and the percentage engaged in vigorous physical activity increased by 26.2% [36]. These increases were sustained at 1 year from baseline. Cheadle et al’s study on a community level ‘Healthy Eating Active Living Zones’ initiative also indicated that positive changes in diet and physical activity within population can be seen when the intervention is delivered to a larger number of people for a longer duration.

### Group medical appointments

There were two studies that incorporated group medical appointments with prevention strategies, such as education and improving self-care measures [45, 46]. One study was high risk of bias [46] while the other one was low risk of bias [45]. Beck et al. evaluated the impact of PHI initiated group medical visits (which included health education, socialisation opportunities, mutual support, and prevention measures) on hospital utilisation and costs for chronically ill older members compared with standard care [45]. They found that after 1 year members in the intervention arm had less emergency department visits (*p* = 0.009), less specialist visits (*p* = −.028) and less hospital admissions (*p* = 0.051), instead they made more nurse calls (*p* = 0.038) and fewer physician calls (*p* = 0.019) than the control group. Average cost saving per participant of the intervention was $14.79. Moreover, after a year, a greater percentage of intervention participants received vaccinations for influenza and pneumonia and had a greater satisfaction with care compared to the control group (81% vs. 64%) [45]. Another program that utilised group medical appointment and education was Operation Zero (O.Z.) in paediatric obesity [46]. At 8 weeks after program completion compared with baseline, there were significant reductions in the percentage of body fat and waist size for those who attended 6 or more sessions. The program showed effectiveness after 6 months for weight maintenance, but not after 1 year. After 1 year, the mean change in weight and BMI in the O.Z. group was not statistically different compared to the control group [46].

## Discussion

In recent years, there has been a growing interest in prevention of chronic diseases within the PHI setting. Many chronic disease prevention interventions have been delivered by PHIs in many countries. However, to the best of our knowledge, a systematic review has not yet been conducted on the impact of these interventions, and this is the first systematic review that explored the impact of variety of chronic disease prevention interventions implemented by PHIs. Twenty-nine studies were included in our final thematic synthesis.

Our results confirmed earlier systematic reviews on chronic disease prevention programs in different settings [15], highlighting that these interventions are generally effective in reducing risk factors of chronic diseases. The studies in our review had evaluated interventions for various outcome measures. While some studies measured healthy behaviour or weight loss as outcomes, other studies measured healthcare utilization or a mix of both. This review showed that the most studies were about health coaching, wellness programs and incentives, which could indicate that these intervention types were the most commonly used by PHIs.

Studies on health coaching found them to be effective in improving healthy eating, physical activity, quality of life, and reducing hospital utilisation [28]. However, the duration of effects was different for each intervention. Incentive programs were shown to increase healthy food consumption, and to achieve a statistically significant difference in self-reported dietary behaviours. However, An et al. concluded that there was no evidence to support the association between Vitality incentive programs for healthy food and reducing BMI or preventing obesity, despite self-reported reductions on BMI [20]. This shows the need for evaluating such programs by using measured BMI as an outcome measure in future studies. Vitality program interventions that were based on activities around fitness, screening and health assessment, education and healthy choices showed evidence of increased use of gym and physical activities. However, more than two-thirds of members still failed to use their gym benefits in a meaningful way for health improvement, suggesting that the incentives and subsidy are not sufficient to overcome behavioural inertia in a majority of members.

Regardless of the intervention types, it appeared that PHI interventions that of longer duration (more than 2 years) were more beneficial and outcomes were more sustainable than those PHI interventions that lasted for a shorter period. For example, incentive programs showed higher achievements in the short term: the 4-month incentive program by GMHBA indicated a statistically significant difference in increase of leisure time physical activities, transport related physical activities, reduced sitting time and BMI at the end of 4 months. However, once incentives ceased, these impacts also reduced or were lost in the long run. This pattern was visible for both health coaching and wellness programs. King et al. also mentioned that a significant healthcare cost reduction due to reduced hospital admission was visible by the end of a 2 year fitness program, but the participation rates were starting to decline in the program’s second year, with only 61% of participants continuing to participate [39].

Group medical appointments also indicated the same pattern of short-term improvements which were harder to sustain. While group medical appointments for weight loss management were shown to be effective after a year in reducing hospital utilisation and costs, and the changes were statistically significant, they were not effective for weight loss management after a year. For example, the O.Z. program was effective after 6 months, but after a year the mean change in weight and BMI of O.Z. participants were no longer statistically different to those of the control group. These results indicate that PHI group medical appointment programs can produce positive outcomes in the shorter term, but do not usually offer sustainable, long-term benefits once the intervention ceases.

The strengths of this systematic review are: the search of both academic and grey literature; and that the search was not limited to one geographical area or population. The limitations include: diversity of the interventions, methodology, and outcome measures of studies made comparability across interventions and outcomes challenging. Studies comparing different intervention types were not found, and the outcomes presented across the studies were varied and not sufficiently comparable to allow any decision on which intervention is the most effective. Many of the included studies were likely to have a high risk of bias, and therefore there is a need for future research to evaluate chronic disease prevention interventions by PHIs to establish their effectiveness and cost effectiveness in real practice settings.

### Implications and conclusion

Chronic disease prevention interventions by PHI, such as the Vitality program, health coaching, wellness programs, financial incentives and group medical appointments, have been shown to generate measurable health benefits for participants. Discovery Health and its Vitality program stand out as having made a particularly innovative contribution both in the design and the evaluation of interventions in this field. The results of this review show that all these approaches to chronic disease prevention by PHIs have been shown to be effective in the short term, but their impacts did not last in the long run once the intervention was ceased. It therefore seems likely that PHI prevention programs must themselves be sustained for long periods if their beneficial outcomes are also to be sustained. Future research should therefore focus on how best to design and deliver programs and interventions to achieve sustained behaviour change over long periods. This research shows that PHI insurers do have the potential to play a significant and beneficial role in improving the health of their members and the wider community, but only if they can take a longer-term perspective and act as “patient investors” in health and well-being.

### Other information

The study protocol was registered in PROSPERO (CRD42020145644) [47] and can be accessed via https://www.crd.york.ac.uk/PROSPERO/. The protocol was amended to include a researcher who contributed to the study and to update the completion date.

## Supplementary Information

**Additional file 1.**

**Additional file 2.**

**Additional file 3.**

**Additional file 4.**

## Data Availability

All data generated or analysed during this study are included in this article and its additional files.
