# Supplementary material for: Impacts of chronic disease prevention programs implemented by private health insurers: a systematic review
Source: BMC Health Serv Res. 2021 Nov 11;21:1222. doi: 10.1186/s12913-021-07212-7 (PMC8582197; doi:10.1186/s12913-021-07212-7)
Supplement: Supplementary file 2 — Additional file 2. [file 12913_2021_7212_MOESM2_ESM.pdf]

Additional File 2: Search Terms

Search terms used for Medline, Business Source Complete, CINAHL, Global Health, Health Business Elite, PsycINFO databases (Limiters: English language and published until 30th September 2020)

S1 “private health insur*” OR “Health maintenance organi?ation*” OR “private healthcare”
S2 "chronic disease*" OR “chronic illness*” OR “long term disease*” OR “chronic condition*”
S3 Prevent*
S4 Program* OR intervention* OR plan* OR coach* OR strateg* OR management
S5 Health N2 (promot* OR program* OR intervent*)
S6 Wellness OR wellbeing N2 (promot* OR program* OR intervent*)
S7 Screening OR vaccin* N2 (promot* OR program* OR intervent*)
S8 “health literac*” N2 (promot* OR program* OR intervent*)
S9 “health reward*” N2 (PROGRAM* OR INCENTIV* OR strate*)
S10 S5 OR S6 OR S7 OR S8 OR S9
S11 S2 AND S3
S12 S10 OR S11
S13 S1 AND S4 AND S12

Search terms used for Scopus database (Limiters: English language and published until 30th September 2020)
S1 “private health insur*” OR “Health maintenance organi?ation*” OR “private healthcare”
S2 "chronic disease*" OR “chronic illness*” OR “long term disease*” OR “chronic condition*”
S3 Prevent*
S4 Program* OR intervention* OR plan* OR coach* OR strateg* OR management
S5 Health W/2 (promot* OR program* OR intervent*)
S6 Wellness OR wellbeing W/2 (promot* OR program* OR intervent*)
S7 Screening OR vaccin* W/2 (promot* OR program* OR intervent*)
S8 “health literac*” W/2 (promot* OR program* OR intervent*)
S9 “health reward*” W/2 (PROGRAM* OR INCENTIV* OR strate*)
S10 S5 OR S6 OR S7 OR S8 OR S9
S11 S2 AND S3
S12 S10 OR S11
S13 S1 AND S4 AND S12

Search terms used for Google and Google Advanced search
Combination of “chronic disease”, Prevent, “private health insur” OR “Health maintenance organization”, intervention OR program
