# Supplementary material for: Impacts of chronic disease prevention programs implemented by private health insurers: a systematic review
Source: BMC Health Serv Res. 2021 Nov 11;21:1222. doi: 10.1186/s12913-021-07212-7 (PMC8582197; doi:10.1186/s12913-021-07212-7)
Supplement: Supplementary file 3 — Additional file 3. [file 12913_2021_7212_MOESM3_ESM.pdf]

Additional File 3: Quality assessment of selected articles

Author/s, Year	Strong	Moderate	Poor
An et al., 2013			
Patel et al., 2011			
Patel et al., 2010			
Sturm et al., 2013			
Hubbert et al., 2003			
Schwartz et al., 2014			
Ball et al., 2017			
Lambert et al., 2009			
McGill et al., 2018			
Adams et al., 2013			
Koocher et al., 2001			
Härter et al., 2013			
Schwartz et al., 2010a			
Scuffham et al., 2019			
Lawson et al., 2013			
Schmittdiel et al., 2017			
Harmar et al., 2010			
Morello et al., 2016			
Harris, 2011			
King et al., 2012			
Maeng et al., 2013			
Henry et al., 2016			
Frost et al., 2018			
Cheadle et al., 2018			
McGill et al., 2020			
Coombes, 1998			
Schwartz et al., 2010b			
Beck et al., 1997			
Hinchman et al., 2006			
Total	1	4	24
