# Supplementary material for: Impacts of chronic disease prevention programs implemented by private health insurers: a systematic review
Source: BMC Health Serv Res. 2021 Nov 11;21:1222. doi: 10.1186/s12913-021-07212-7 (PMC8582197; doi:10.1186/s12913-021-07212-7)
Supplement: Supplementary file 4 — Additional file 4. [file 12913_2021_7212_MOESM4_ESM.pdf]

## Additional File 4: Summary of the outcomes

| Author, year | Program | Outcome measure | Outcome in detail | Results | Effect measure |
|---|---|---|---|---|---|
| Studies on financial incentives | | | | | |
| An et al., 2013 | Cash-back for healthy food program | Self-reported measures of food consumption and weight status (BMI) | Increase in daily fruits and vegetables consumption by (10% discount)<br>Increase in daily fruits and vegetables consumption by (25% discount)<br>More likely to have three or more servings of wholegrain foods daily (10% discount)<br>More likely to have three or more servings of wholegrain foods daily (25% discount)<br>Less likely to regularly have foods high in sugar (10% discount)<br>Less likely to regularly have foods high in sugar (25% discount)<br>Less likely to regularly have foods high in salt (10% discount)<br>Less likely to regularly have foods high in salt (25% discount)<br>Less likely to regularly have fried food (10% discount)<br>Less likely to regularly have fried food (25% discount)<br>Less likely to regularly have processed food (10% discount)<br>Less likely to regularly have processed food (25% discount)<br>Less likely to regularly have fast food (10% discount)<br>Less likely to regularly have fast food (25% discount)<br>Statistically significant association between lower obesity and 25% discount | 0.38 (servings)<br>0.64 (servings)<br>2.05 (OR)<br>2.96 (OR)<br>0.73 (OR)<br>0.35 (OR)<br>0.59 (OR)<br>0.26 (OR)<br>0.53 (OR)<br>0.26 (OR)<br>0.71 (OR)<br>0.33 (OR)<br>0.54 (OR)<br>0.28 (OR)<br>0.86 (OR) | (95% CI: 0.37 – 0.39)<br>(95% CI: 0.62 – 0.65)<br>(95% CI: 1.97 – 2.13)<br>(95% CI: 2.84 – 3.08)<br>(95% CI: 0.69 – 0.76)<br>(95% CI: 0.34 – 0.37)<br>(95% CI: 0.55 – 0.62)<br>(95% CI: 0.25 – 0.28)<br>(95% CI:0.50 – 0.55)<br>(95% CI: 0.25 – 0.27)<br>(95% CI: 0.68 – 0.74)<br>(95% CI: 0.31 – 0.34)<br>(95% CI:0.51 – 0.56)<br>(95% CI: 0.27 – 0.29)<br>(95% CI: 0.81 – 0.91) |
| Patel et al., 2011 | Vitality health promotion program | Changes in the participation in activities on fitness, and hospital admissions | Percentage of inactive (years 1-5)<br>Percentage of low-active (years 1-5)<br>Percentage of medium-active (years 1-5)<br>Percentage of high-active (years 1-5)<br>Probability of hospital admissions per number of additional gym visits per week<br>1+ every 2 weeks<br>1+ per week<br>3+ every 2 weeks<br>2+ per week | 0.58 (OR)<br>1.04 (OR)<br>1.63 (OR)<br>1.26 (OR)<br><br>0.97 (OR)<br>0.93 (OR)<br>0.90 (OR)<br>0.87 (OR) | (95% CI: 0.57-0.58)<br>(95% CI: 1.02-1.07)<br>(95% CI: 1.6-1.65)<br>(95% CI: 1.25-1.28)<br><br>(95% CI: 0.95-0.97)<br>(95% CI: 0.90-0.93)<br>(95% CI: 0.85-0.90)<br>(95% CI: 0.80-0.87) |
| Patel et al., 2010 | Vitality physical activity | Hospital admissions | Admission rate per member (Not registered) compared to others<br>Admission rate per member (Not Engaged) compared to others<br>Admission rate per member (Lowly Engaged) compared to others<br>Admission rate per member (Highly Engaged) compared to others<br>Difference in admission rates between highly engaged and others (cancer)<br>Difference in admission rates between highly engaged and others (cardiovascular diseases)<br>Difference in admission rates between highly engaged and others (endocrine and metabolic)<br>Difference in admission rates between highly engaged and others (musculoskeletal) | 0.42 (p<0.001)<br>0.39 (p n.s.)<br>0.46 (p<0.001)<br>0.39 (p n.s.)<br>-13.17% (p<0.01)<br>-7.37% (p<0.0001)<br>-20.66% (p<0.0001)<br>-15.60% (p<0.0001) | (95% CI: 0.419-0.425)<br>(95% CI: 0.388-0.396)<br>(95% CI: 0.459-0.466)<br>(95% CI: 0.382-0.393)<br>(95% CI: -3.57%- -22.63%)<br>(95% CI: -5.49%- -9.35%)<br>(95% CI: -14.83%- -26.53%)<br>(95% CI: -10.67%- -20.55%) |
| Sturm et al., 2013 | Cash-back for healthy food | Changes in healthy food purchases | Increase in the ratio of healthy to total food expenditure (10% rebate)<br>Increase in the ratio of healthy to total food expenditure (25% rebate)<br>Increase in the ratio of fruit and vegetables to total food expenditure (10% rebate)<br>Increase in the ratio of fruit and vegetables to total food expenditure (25% rebate)<br>Decrease in the ratio of less desirable to total food expenditure (10% rebate)<br>Decrease in the ratio of less desirable to total food expenditure (10% rebate) | 6.0%<br>9.3%<br>5.7%<br>8.5%<br>5.6%<br>7.2% | (95% CI: 5.3 - 6.8)<br>(95% CI: 8.5- 10.0)<br>(95% CI: 4.5- 6.9)<br>(95% CI: 7.4- 9.7)<br>(95% CI: 4.7- 6.5)<br>(95% CI: 6.3- 8.1) |

| Author, year | Program | Outcome measure | Outcome in detail | Results | Effect measure |
|---|---|---|---|---|---|
| Hubbert et al., 2003 | Financial incentives on a weight loss program | Participation rate and weight loss | Mean attendance (no. of classes) (with incentives)<br>Mean attendance (no. of classes (without incentives)<br>Proportion (%) attending ≥10 classes (with incentives)<br>Proportion (%) attending ≥10 classes (without incentives)<br>Proportion (%) losing ≥% body weight (with incentives)<br>Proportion (%) losing ≥6% body weight (without incentives)<br>Proportion attending ≥10 classes and losing ≥6% body weight (with incentives)<br>Proportion attending ≥10 classes and losing ≥6% body weight (without incentives) | 10.1± 1.8<br>8.2± 2.5<br>84%<br>37%<br>56%<br>20%<br>56%<br>14% | P<0.001<br>P<0.001<br>P<0.001<br>P<0.001<br>P<0.001<br>P<0.001<br>P<0.001<br>P<0.001 |
| Schwartz et al., 2014 | Financial incentives | Healthy food purchases | Percentage Increase in healthy grocery item purchases | 3.5% | |
| Ball et al., 2017 | The ACHIEVE (Active Choices IncEntiVE) | Changes in physical activity and sedentary behaviour | Mean (SD) leisure time physical activity (mins/week) (baseline vs. follow-up)<br>Mean (SD) transport related physical activity (mins/week) (baseline vs. follow-up)<br>Mean (SD) sitting (hours/day) (baseline vs. follow-up) | 92.0 (118.5) vs. 344.5 (406.6)<br>77.9 (90.7) vs. 256.8 (318.2)<br>8.5 (2.5) vs. 5.4 (2.0) | P<0.001, Effect size 0.61<br>P<0.001, Effect size 0.56<br>P<0.001, Effect size 1.35 |
| Lambert et al., 2009 | Vitality – incentive based physical activity | Hospital admissions and costs | Mean no. of admissions per member (not registered)<br>Mean no. of admissions per member (inactive)<br>Mean no. of admissions per member (Low active)<br>Mean no. of admissions per member (Moderate active)<br>Mean no. of admissions per member (High active)<br>Mean cost per member (Not registered)<br>Mean cost per member (inactive)<br>Mean cost per member (Low active)<br>Mean cost per member (Moderate active)<br>Mean cost per member (High active) | 0.42<br>0.44<br>0.42<br>0.41<br>0.36<br>8,644 (2006 South Africa Rand)<br>9,075 (2006 South Africa Rand)<br>8,770 (2006 South Africa Rand)<br>8,642 (2006 South Africa Rand)<br>7,540 (2006 South Africa Rand) | (95% CI: 0.42-0.42)<br>(95% CI: 0.44-0.44)<br>(95% CI: 0.42-0.43)<br>(95% CI: 0.40-0.42)<br>(95% CI: 0.36-0.37)<br>(95% CI: 8,545-8,743)<br>(95% CI: 8,966-9,184)<br>(95% CI: 8,560-8,980)<br>(95% CI: 8,428-8,856)<br>(95% CI: 7,354-7,727) |
| McGill et al., 2018 | Australian weight loss and lifestyle modification program (Financial incentives) | Attitudes and views of participants | Percentage of participants who believed that 3-6 months incentive after a program would be helpful in weight loss maintenance<br>Percentage who perceived non-cash incentives would motivate weight loss maintenance<br>Percentage who perceived that cash incentives would motivate weight loss maintenance<br>Percentage who perceived that deposit contracts would motivate weight loss maintenance<br>Percentage who perceived that matched deposit contracts would motivate weight loss maintenance | 56.9%<br>85.3%<br>77%<br>40.5%<br>48.7% | Not applicable |
| Studies on health coaching | | | | | |
| Adams et al., 2013 | Health coaching | Patient satisfaction and perceived success | Percentage of those who were satisfied with the program<br>Percentage of those who would recommend health coaching<br>Percentage of those who were neutral about the program<br>Percentage of self-reported respondents who attended 2 or more session compared to 1 session:<br>Eat more healthfully<br>Increase physical activity<br>Improve health<br>Improve quality of life<br>Reduce risk of diseases | 70%<br>71%<br>20%<br><br>68% vs. 54%<br>71% vs. 54%<br>79% vs. 61%<br>83% vs. 61%<br>73% vs. 51% | <br><br><br><br>P=0.04<br>P<0.001<br>P=0.005<br>P<0.001<br>P=0.10 |

| Author, year | Program | Outcome measure | Outcome in detail | Results | Effect measure |
|---|---|---|---|---|---|
| Koocher et al., 2001 | Medical crisis counselling | Reductions in distress, hospital utilisation and costs | Reduction in anxiety scale for cancer patients who received the program<br><br>Reduction in anxiety scale for cardiac patients who received the program<br><br>No increases in overall medical costs | t statistic (36) = 2.107<br>anxiety scale- n.a.<br>t statistic (23) =2.196<br>anxiety scale- n.a. | P=0.043<br><br>P=0.04 |
| Härter et al., 2013 | Health coaching | Evaluation of the coaching and process, effects on patient and physician communication | Percentage of people who were satisfied with the coaching<br>Percentage of people who would recommend the coaching to others<br>Percentage of people who learnt new options to influence health conditions<br>Percentage of people who found the information was comprehensive<br>Percentage of people who found the amount of information adequate<br>Percentage of people who found the coaching as useful | 78.3%<br>82.3%<br>53.3.%<br>84.8%<br>81.2%<br>67.9% | Not applicable |
| Schwartz et al., 2010a | Online disease management program with coaching | Medical costs and claims | Health care costs per person per year<br>Return on investment for every dollar spent on the program | 757 (2008 US$)<br>9.89 (2008 US$) | n.a. |
| Scuffham et al., 2019 | Health coaching for disease management (CAPICHe) | Healthcare utilisation and costs | Mean estimated total cost 1-year post-intervention (intervention group)<br>Mean estimated total cost 1-year post-intervention (control group)<br>Same-day admission cost (intervention group)<br>Same-day admission cost (group group)<br>Same-day admissions per 1000 person-years (intervention group)<br>Same-day admissions per 1000 person-years (control group) | 4934 (2012AUS$)<br>4868 (2012AUS$)<br>468 (2012AUS$)<br>508 (2012AUS$)<br>530<br>614 | (95% CI: AUS$4823–5045)<br>(95% CI: AUS$4680-5058)<br>(95% CI: 454-482)<br>(95% CI: 484-533)<br>(95% CI: 508-552)<br>(95% CI: 571-657) |
| Lawson et al., 2013 | Health coaching-telephonic | Health and behavioural outcomes | Percentage of people who met at least one lifestyle change<br>Increase motivation among participants to change by<br>Increase confidence among participants in ability to change<br>Increase readiness to make change<br>Increase in limiting fat in foods<br>Increase in being very satisfied with physical activity level | 89%<br>11%<br>15%<br>34%<br>12.8%<br>20.9% | Not applicable |
| Schmittdiel et al., 2017 | Wellness coaching on weight loss | Changes in weight loss | BMI trajectory after start of wellness coaching compared to baseline<br>BMI trajectory for control group compared to baseline | -1.79 kg/m$^2$ (negative)<br>-0.26 kg/m$^2$ (no change) | P<0.001<br>P=0.11 |
| Harmar et al., 2010 | Telephonic coaching in Chronic care management program | Hospital admission rate | Decrease in admission rate (intervention group)<br>Increase in admission rate (control group) | 6.2%<br>14.9% | <br>P<0.001 |
| Morello et al., 2016 | Telephonic Complex Care Program | Hospital use and benefits paid | Mean difference of hospital admissions after 12 months to base line (intervention group)<br>Mean difference of hospital admissions after 12 months to base line (control group)<br>Increase in benefits paid for ancillary claims made per intervention participant in 12 months | 2.4 (p<0.005)<br>2.3 (p<0.005)<br>120.45 (2013AUS$) | (95% CI 1.1-3.6)<br>(95% CI: 0.8-3.8) |
| Studies on wellness programs | | | | | |
| Harris, 2011 | Wellness program (Healthy | Medical utilisation, health | Mean (SD) number of inpatient admissions in 2006 (program continuers vs non-continuers)<br>Mean (SD) number of emergency department visits in 2006 (program continuers vs non-continuers) | 0.06 (0.25) vs. 0.06 (0.32)<br>0.10 (0.39) vs. (0.12 (0.43) | P=0.436<br>P=0.053 |

| Author, year | Program | Outcome measure | Outcome in detail | Results | Effect measure |
|---|---|---|---|---|---|
| | Lifestyle rewards program) | claims, health risk behaviours | Mean (SD) claims on depression in 2006 (program continuers vs non-continuers)<br>Mean (SD) Change in smoking status attributable to intervention- Smoke free help | 0.16 (0.36) vs. 0.15 (0.36)<br>-0.585 (0.078) | P=0.722 |
| King et al., 2012 | Health-plan sponsored fitness centers | Changes in physical activity and positive health outcomes, health utilisation | Frequency of fitness centre visits declined in year 2 of a program (year 1 vs year 2)<br>Median for year 1 (Interquartile range)<br>Median for year 2 (Interquartile range)<br>Decrease in total health care costs by the end of year 2<br>Adjusted healthcare cost for those with 2 to <3 visits to fitness centre<br>Adjusted healthcare cost for those with ≥ 3 visits to fitness centre | 75 vs. 55<br>49 (11-1120)<br>12 (0-89)<br>-500 (US$ year n.a.)<br>-1252 (US$ year n.a.)<br>-1309 (US$ year n.a.) | <br><br><br>(CI: -$892- -$106), p=0.01<br>P<0.001<br>P=0.001 |
| Maeng et al., 2013 | MyHealth Rewards (health plan-driven employee health and wellness program) | Impact on cardiovascular event rate and health costs | Experience of stroke (hazard ratio)<br>Experience of myocardial infraction (hazard ratio)<br>Percentage of cost reduction during the $2^{nd}$ and $3^{rd}$ year of the program<br>Return on investment of the program | 0.73<br>0.56<br>10-13%<br>1.52 (-1.99- to 5.02) | (95% CI: 0.60-0.90)<br>(95% CI: 0.40-0.79) |
| Henry et al., 2016 | Internet based health promotion | Reducing care gaps on haemoglobin A1c testing, pneumonia vaccination, and cancer screenings | Adjusted Odds of Care Gap Closure by the program users compared to non-users<br>HbA1c testing<br>Pneumonia vaccine<br>Mammogram<br>Pap smear<br>Colorectal cancer screening | <br>1.12<br>0.95<br>1.09<br>1.06<br>1.09 | <br>(95% CI: 1.06-1.18)<br>(95% CI: 0.91-1.00)<br>(95% CI: 1.05-1.14)<br>(95% CI: 1.02-1.11)<br>(95% CI: 1.06-1.12) |
| Frost et al., 2018 | Physical activity | Participation in physical activity and changes in physical activity | Increase in % of children engaged in moderate to vigorous physical activity in 6 months<br>Increase in % of children engaged in vigorous physical activity in 6 months<br>Increase in % of children engaged in moderate to vigorous physical activity at 1 year from baseline<br>Increase in % of children engaged in vigorous physical activity at 1 year from baseline | 23.3%<br>26.2%<br>17.2%<br><br>33.1% | |
| Cheadle et al., 2018 | Healthy eating, Active living | Changes in food and physical activity behaviours | Pre and post increases in moderate or rigorous physical activity<br>Number of restaurants that had made a healthy menu after 6 months (out of 10 restaurants in one community) | 17% vs. 19%<br>Only 3 | |
| McGill et al., 2020 | The Healthy Weight for Life (HWFL) | Changes in anthropometric and lifestyle risk behaviours | Percentage of participants who achieved the weight loss maintenance at 3 months<br>Percentage of participants who achieved the weight loss maintenance at 6 months<br>Percentage of participants who achieved the weight loss maintenance at 9 months<br>Percentage of participants who achieved the weight loss maintenance at 12 months<br>Mean change in physical activity (minutes/week)<br>Mean change in sitting (minutes/day)<br>Mean change in healthy eating (vegetables serves/day)<br>Mean change in healthy eating (fruits serves/day) | 76%<br>62%<br>55%<br>56%<br>-56.5<br>+24.6 (16.9)<br>+0.4 (0.2)<br>+0.1 (0.1) | <br><br><br><br>P=0.059<br>P=0.147<br>P=0.009<br>P=0.383 |
| Coombes, 1998 | Malnutrition screening | Identification of risk patients | Percentage of patients with high nutritional risk<br>Percentage of patients with moderate nutritional risk | 22%<br>30% | |

| Author, year | Program | Outcome measure | Outcome in detail | Results | Effect measure |
|---|---|---|---|---|---|
| Schwartz et al., 2010b | wellness and disease prevention program (HealthPass) | Healthcare costs | Return on investment for every dollar invested for years of 2002-2005 | 2.83 (2002US$)<br>1.16 (2003US$)<br>1.56 (2004US$)<br>1.58 (2005US$) | |
| Studies on group medical appointments | | | | | |
| Beck et al., 1997 | Group outpatient medical appointment | Health service utilisation and cost, self-reported health status, and satisfaction | Mean Emergency room visits (intervention vs. control group)<br>Visits to subspecialists (intervention vs. control group)<br>Visits and calls to nurses (intervention vs. control group)<br>Satisfaction with care (intervention vs. control group)<br>Aggregated cost savings<br>Cost of care per member per month | 0.41 vs. 0.67<br>3.22 vs. 3.95<br>8.7 vs. 7.89<br>49% vs. 27%<br>31,928 (1997US$)<br>14.79 (1997US$) | P=0.009<br>P=0.028<br>P=0.038<br>P=0.019 |
| Hinchman et al., 2006 | Childhood obesity | Body fat reduction, waist size, BMI | Mean (SD) change in waist size for those who attended ≥ 6 sessions<br>Mean (SD) change in body fat for those who attended ≥ 6 sessions<br>Mean (SD) change in waist size for preteens who attended ≥ 6 sessions<br>Mean (SD) change in body fat for preteens who attended ≥ 6 sessions<br>Mean (SD) change in waist size for adolescents who attended ≥ 6 sessions<br>Mean (SD) change in body fat for adolescents who attended ≥ 6 sessions<br>Mean (SD) change of weight (lb) after 6 months from baseline (intervention group vs. control group)<br>Mean (SD) change of BMI ($kg/m^2$) after 6 months from base line (intervention group vs. control group)<br>Mean (SD) change of BMI (%) after 6 months from baseline (intervention group vs. control group) | -0.55 (1.41) (inches)<br>-1.63 (4.39) (%)<br>-1.21 (1.40) (inches)<br>-1.68 (4.09) (%)<br>-0.70 (1.57) (inches)<br>-0.58 (1.61) (%)<br>2.35 (9.90) vs. 7.64 (10.00)<br>-1.06 (2.27) vs. 0.79 (1.67)<br>-0.10 (0.22) vs. -0.36 (1.11) | P<0.05<br>P<0.05<br>P<0.05<br>P<0.05<br>P<0.05<br>P<0.05<br>P n.sn (intervention group) & p<0.05 (control group)<br>P n.s. for both groups<br>P n.s. for both groups |

(Notes: OR -Odd Ratio, CI confidence interval, BMI Body Mass Index, SD Standard Deviation, n.s. Not Significant, n.a. Not Available)
